# Prognosis of extremely severe lactic acidosis in metformin-treated patients with septic shock: continuous (?) renal replacement therapy in the spotlight!

**DOI:** 10.1186/s13054-016-1286-5

**Published:** 2016-05-07

**Authors:** Patrick M. Honore, Herbert D. Spapen

**Affiliations:** ICU Department, Universitair Ziekenhuis Brussel, Vrije Universiteit Brussel, 101, Laarbeeklaan, Jette, Brussels 1090 Belgium

We read with interest the recent paper by Doenyas-Barak et al. [1] comparing the prognosis of extremely elevated plasma lactate levels between metformin (MET)-treated and MET-naive patients with septic shock. The most remarkable finding was a highly significant lower in-hospital mortality (56.8 vs. 88.1 %, *p* < 0.0001) in MET users despite a more explicit risk profile (diabetes, older age) and greater baseline disease severity (higher incidences of cardiovascular disease, acute kidney injury, and underlying malignancy) [1]. The authors briefly elaborate on this unexpected survival benefit by pointing out some potential protective MET-related anti-inflammatory, anti-thrombotic, and cellular effects. However, we believe that the observed difference in mortality rate is most likely due to the more frequent use of renal replacement therapy (RRT) in the MET-treated population (38.6 vs. 21.2 %, *p* = 0.13).

Whereas initiation of RRT is generally stigmatized as a bad prognostic sign in the critically ill, it might be life-saving in MET users presenting with septic shock and severe lactic acidosis. A protective effect of RRT has already been suggested by Peters et al. [2] who found that, despite higher illness severity, the mortality rate in patients with MET-associated lactic acidosis treated with intermittent hemodialysis (IHD) was no different to that of non-dialyzed subjects. Experts recommend RRT at lactate concentrations >20 mmol/L or pH ≤7.0, in case of shock or decreased level of consciousness, and when standard supportive measures fail [3]. Early IHD is the preferred mode of treatment. For several reasons, however, continuous RRT (CRRT) is thought to be physiologically more appropriate than IHD. First, because of its low molecular weight and minimal protein binding, MET is equally (highly) eliminated by ultrafiltration (convection) as compared to dialysis (diffusion). Second, and more importantly, its large volume of distribution within a two-compartment pharmacokinetic model implies that MET may be more effectively cleared by prolonged RRT. This was corroborated by Keller et al. [4] who recently showed a dramatic reduction of metabolic acidosis and decrease of MET plasma concentrations within the first 24 h after initiating CRRT in patients with MET-induced lactic acidosis, followed by normalization on the second day in all subjects. Finally, consensus exists that CRRT is better tolerated in hemodynamically unstable patients, and is also associated with a higher rate of renal recovery compared with IHD [5]. This will certainly benefit septic MET-imbedded patients who often present with catecholamine-dependent septic shock and faltering or lost kidney function.

## Abbreviations

CRRT: continuous renal replacement therapy
HR: hazard ratio
IHD: intermittent hemodialysis
MALA: metformin-associated lactic acidosis
MET: metformin
RRT: renal replacement therapy
