# Supplementary material for: Prognosis of extremely severe lactic acidosis in metformin-treated patients with septic shock: continuous (?) renal replacement therapy in the spotlight!
Source: Crit Care. 2016 May 7;20:127. doi: 10.1186/s13054-016-1286-5 (PMC4859991; doi:10.1186/s13054-016-1286-5)
Supplement: Related texts

# Reply: Authors’ response

**Authors:** K. Doenyas-Barak, I. Beberashvili, S. Efrati

The role of RRT in the treatment of lactic acidosis in general, and in metformin-associated lactic acidosis (MALA) in particular, has been the subject of several trials. Due to the low molecular weight of lactate (90 Da, which is very similar to urea), and its easy removal by RRT, patients with severe lactic acidosis are good candidates for RRT [6]. However, lactate itself is not toxic, and data regarding the yield of RRT in patients with severe lactic acidosis are scarce. In MALA, RRT may contribute to both lactate and metformin removal. Unfortunately, data regarding the effect of RRT in the setting of MALA are also limited, being based mostly on observational studies or very small trials. In one of them, although improved prognosis of patients requiring RRT was not actually demonstrated, the authors believed that the prognosis might have been worse [2]. However, even though reliable data comparing RRT given "prophylactically" at an early stage vs. RRT given according to classical clinical indications (as determined by the treating nephrologist) do not exist, some experts still strongly recommend the earliest possible initiation of RRT [3].

Following the letter of Drs. Honore and Spapen, we performed further statistical analysis of our study cohort to evaluate whether more frequent RRT in the metformin-treated group may explain the survival benefit. Multivariate Cox regression analyses were redone for the evaluation of adjusted hazard ratios (HRs) of metformin treatment, with RRT added to the confounders (in addition to age, gender, diabetes, hypertension, ischemic heart disease, cerebrovascular attack, malignancy, creatinine, and Acute Physiology and Chronic Health Evaluation (APACHE) II score). The effect of the hazard related to metformin treatment remained statistically significant (HR = 0.18; 95 % confidence interval 0.03–0.92; *p* = 0.04). Thus, the use of RRT does not appear to explain the protective effect of metformin in the current cohort of patients [1].

Another unresolved issue relates to the preferred RRT modality, and to whether CRRT may be physiologically more appropriate than IHD for MALA patients. CRRT is the preferred modality in patients who are hemodynamically unstable and cannot tolerate IHD due to blood pressure fluctuations. However, for other MALA patients, hemodialysis may be the preferred approach since metformin clearance is less effective under the convective treatment used in CRRT [7]. Unfortunately, the size of our study population was not sufficient to address the preferred RRT modality for MALA patients.
